# Question prompt lists and endorsement of question‐asking support patients to get the information they seek—A longitudinal qualitative study

**DOI:** 10.1111/hex.13509

**Published:** 2022-04-26

**Authors:** Marguerite Tracy, Julie Ayre, Olivia Mac, Tessa Copp, Emerita Lyndal Trevena, Heather Shepherd

**Affiliations:** ^1^ ASK‐GP Centre for Resarch Excellence Sydney School of Public Health, The University of Sydney New South Wales Australia; ^2^ Susan Wakil Sydney Nursing School The University of Sydney New South Wales Australia

**Keywords:** doctor endorsement, health literacy, health services accessibility, patient participation, question prompt lists, shared decision‐making

## Abstract

**Introduction:**

Question prompt lists (QPLs) have been found to support patients to ask questions and improve the information they receive from doctors. However, some QPL tools, which have been available online for almost 15 years, have little published data on their impact in real‐world settings. This study's aim was to understand patients' attitudes and experiences accessing health information and to assess the impact of introducing two generic QPLs over 3 months.

**Methods:**

A longitudinal qualitative study consisting of three semi‐structured interviews over a three‐month period was conducted with 31 purposively selected participants, adults ≥18 years, recruited online and through social media. Participants were introduced to two different QPLs currently available online (‘Question Builder’; ‘AskShareKnow’). Inductive thematic analysis of a total of 92 semi‐structured telephone interviews was conducted during May–November 2020.

**Results:**

Three main themes are described. (1) Participants described barriers and facilitators to accessing health information: navigating a complex health system; difficulty asking questions of their healthcare professionals and that they value doctors with good communication skills. (2) QPLs helped some participants recognize the role of question‐asking in consultations, made them feel more empowered and helped them prepare and prioritize. (3) Participants wanted QPLs to be easier to use, be accessible when needed and that question‐asking and QPLs should be normalized in medical consultations.

**Conclusions:**

Well‐designed and easily accessible QPLs can empower people to ask questions and be more involved in decisions about their health care. Endorsement of question‐asking in consultations by healthcare professionals and providing QPL tools at the point of contact with health services will be key to realizing the potential of QPLs.

**Patient or Public Contribution:**

This study was completed in conjunction with a reference group consisting of a consumer representative, representatives from the Australian Commission on Safety and Quality in Health Care, Healthdirect Australia Ltd., and the research team.

## 1 INTRODUCTION

The right to ask questions and be informed as a patient are seen as being best practice care and are important for patient engagement and safety.^1^, ^2^, ^3^, ^4^ The asking of questions is an important part of helping consumers to obtain information and participate in decisions about their health care.^5^, ^6^ Healthcare professionals (HCPs) should make it clear to the patient when there is a decision to be made about their health care, explain the benefits and harms of the options available, discuss the preferences of the patient and discuss the extent to which the patient wants to make the decision or have the HCP make the decision, either at that time or in the future. These are the core components of the process known as shared decision‐making (SDM) for which there are numerous models and definitions.^7^, ^8^ There are many strategies and tools, including patient decision aids^6^, ^9^ and charters of health rights^10^, ^11^ that have been developed to encourage patient engagement in health care and support SDM. In addition, tools designed to assist people to ask questions during health consultations, known as question prompt lists (QPLs), have been shown to increase question‐asking and improve the information delivered by HCPs and may also facilitate SDM.^12^

There is an abundance of disease‐specific and other QPLs, which are not specific to a particular problem (the latter we will refer to as generic QPLs) available to assist health care consumers to ask questions when they see their HCP.^13^ Literature reviews on QPLs across clinical settings show that they increase question‐asking and improve the amount and quality of information provided by doctors.^12^, ^14^, ^15^, ^16^ Increased quality of information provided was also found with the use of a generic three‐question QPL, AskShareKnow (ASK) questions.^17^ ASK questions have also been shown to be successfully recalled by people with inadequate levels of health literacy who generally ask fewer questions during health consultations,^18^ and also increased their interest in participating in decisions about their health care.^19^ The three ASK questions are: (1) What are my options? (including wait and watch); (2) What are the possible benefits and harms of those options? and (3) How likely are each of the benefits and harms to happen to me?

Another generic QPL is ‘Question Builder’ (QB) developed by the United States' Agency for Healthcare Research and Quality (AHRQ) in 2007.^20^ QB guides users to create a list of questions that can be applied to most health issues. The Australian adaptation of QB contains 101 unique questions and there is a total of 191 questions available across five different appointment types. Some of the 101 questions are repeated under different appointment types. For example, ‘Do I need any tests?’ appears in all three lists for appointments for a general practitioner (‘Routine check up [sic]’, ‘New symptoms’ and ‘Follow‐up’) and both specialist type appointments (‘First visit’ and ‘Follow‐up’).^21^ There are no published data on the use of QB by patients. Importantly, QPLs are most effective when endorsed by the doctor,^12^ which is more likely where they are feasible in real‐world settings.^22^

Despite these potential benefits, data are lacking on the acceptability and usability of generic QPLs by patients in real‐world settings. Up to half of the patients offered a QPL will make use of it but there are few published data to explain the reasons why someone does or does not use a QPL.^12^ Research has shown that while the questions in QPL tools are often created with good accessibility, the instructions are likely to be at a higher reading ability level.^13^

In addition, Australian Government health literacy survey data show that 12% of people reported finding it difficult to engage with healthcare providers.^23^ Lower health literacy correlates with poorer health outcomes;^24^ however, those with lower health literacy perceive QPL interventions to be useful.^19^ Using a patient‐centred approach to address these issues, and qualitative patient‐reported outcomes,^25^ we explore patient experiences through real‐world patient journeys of those with different levels of the adequacy of health literacy as they are introduced to existing generic QPLs and use them in health care consultations. This study's aim was to understand patients' attitudes and experiences accessing health information and assess their perspectives and experiences using two generic QPLs introduced to them over a 3‐month period.

## 2 MATERIALS AND METHODS

### 2.1 Study design

**Figure 1:**
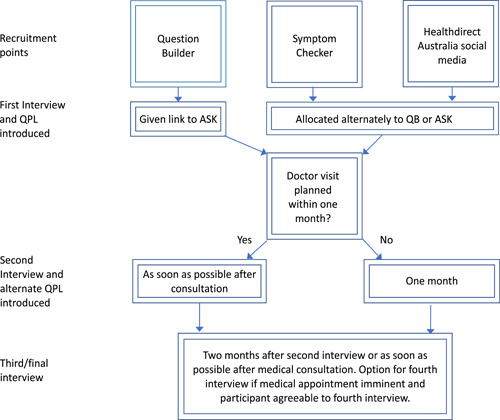
Study design. QB, Question Builder; QPL, question prompt list

This study is a longitudinal qualitative study using online survey recruitment with qualitative data collected through telephone interviews. Participants were invited to participate in a survey and research interviews about ‘how you get the health information you need or want’. Interview participants were invited to complete a series of ideally three interviews over a 3‐month period. Following the first and second interviews, they were introduced to one of two generic QPLs, assigned alternately to QB^21^ or ASK questions^26^ to ensure we had approximately equal numbers of participants exposed to each of the tools. The longitudinal aspect of the study allowed participants to have time with each tool and increase the chance that they would be able to use them in a consultation^17^, ^26^ (see Figure 1 below).

### 2.2 Recruitment survey

Recruitment was via advertisements on the Healthdirect.gov.au website and the Healthdirect Australia Facebook page. Healthdirect is a ‘government‐owned, not‐for‐profit organization’ providing ‘virtual health services’ to consumers.^27^ We chose locations on the Healthdirect website, which corresponded to where consumers were accessing health information, or asking questions about their health, to place recruitment advertisements for participation in the study. Advertisements were placed on the home page of QB and in the ‘Symptom Checker’ tool. Symptom Checker takes users through a series of questions about their symptoms before making a recommendation for action. Interested participants completed an eligibility survey using an online survey system REDCap,^28^, ^29^ with items covering demographic information, health literacy level (using the Chew et al.,^30^ Basic Health Literacy Score [BHLS] screening tool)^30^ and patient activation (Consumer Health Activation Index [CHAI] score)^31^ as an assessment of ‘willingness and ability to take independent actions to manage their health and care’.^32^ These data were used to purposively select participants to achieve a diverse study sample.

### 2.3 Study sample

Our purposive sample was designed to include people with a broad range of demographics, health literacy scores and activation. Our chosen sample aimed to align as closely as possible with Australian population demographics rather than the demographics of the survey sample to ensure adequate representation across states and territories, indigenous self‐identification and health literacy levels. Interviewees were purposively selected from survey responders using participant characteristics (Table 1) to a minimum of 20 participants or until data saturation was reached in the initial interviews.

**Table 1:** Characteristics for purposive sampling

Characteristic	Characteristic options/groups		
Recruitment point	Symptom Checker	Question Builder	Healthdirect Facebook
Age range (years)	18–39	40–59	Over 60
Identified gender	Male	Female	Other
Indigenous self‐identification	Aboriginal	Torres Strait Islander	Does not identify as Indigenous
Level of patient activation (CHAI score)^a^	Low	Moderate	High
Health literacy score^b^	≤11	12‐13	≥14
Language spoken at home	Language other than English	English	
Chronic health condition^c^	Yes	No	
Carer^d^	Yes	No	

^a^ Consumer Health Activation Index (CHAI) score (low: 0–79, moderate: 80–94, high: 95–10 ‘Patients who scored at or below 79 on the CHAI had a nearly three times worse physical decline over 3 years, compared with those scoring 95 and above on the CHAI. Adults scoring between 80 and 94 on the CHAI had over two times worse physical decline over 3 years).^31^

^b^ Chew et al.,^30^ Basic Health Literacy Score (BHLS) screening tool, low health literacy defined as score ≤9.

^c^ Participant self‐identified as having a condition lasting more than 3 months in the participant survey (‘Do you have a health condition which has lasted more than 3 months?’).

^d^ Participant self‐identified as being a carer in the participant survey (‘Do you have someone who is dependent on you for their daily care needs?’).

### 2.4 Interview data collection

We conducted three semi‐structured interviews via telephone with each participant over a 3‐month period. The interview guide was based on previous literature,^12^ and asked about participants' experiences of seeking health information and asking doctors questions (Appendix SA). Following the first and second interviews, participants were emailed a link to either QB or ASK (assigned alternately). Both QPL tools are available in the public domain, they were introduced to participants with a comment at the end of the first and second interviews, such as ‘I'm going to send you an email with a link to a tool called QB/ASK which is about helping people to ask questions when they see the doctor’. The email contained a statement: ‘Here is the link to the QB (or ASK) resource I mentioned. Please take a look before your medical appointment or our next interview’, no further description or instructions were provided. Two participants required additional support via email to use the link provided.

The second and third interviews followed 1 and 2 months after the first, or as soon as possible after a medical consultation with greater emphasis on discussing the participant's QPL thoughts and experiences. Participants were asked at the end of each interview about upcoming or planned health care appointments to enable scheduling of study interviews as soon as possible following these appointments. Participants were encouraged to contact the study team if they had an earlier medical appointment. Where there was no medical appointment, planned or otherwise, the follow‐up interviews took place at the designated study interval, 1 or 2 months following the first or second interviews, respectively.

Participants received a $20 shopping voucher following each interview and could withdraw from the study at any time. Ethics approval was granted by the University of Sydney Human Research Ethics Committee (2019/1015).

### 2.5 Analysis

All interviews were audio‐recorded and transcribed verbatim. Two authors (M. T. and J. A.) conducted an initial inductive analysis by double coding three interviews of two participants and then created initial codes using NVivo software.^33^ All authors reviewed initial codes, coded transcripts and discussed and agreed on themes. The team met three times during coding to refine codes and themes and several times following completion of coding to refine the themes and exemplar data. The analysis followed procedures described by Braun and Clarke.^34^

### 2.6 Reflexivity statement

One of the novel features of this study was the longitudinal approach. M. T. conducted all interviews for this study and took the role of interviewer as a researcher, general practitioner, patient and carer. Pre‐interview assumptions were that there would be: a variety of experiences participants would describe of asking questions to gain information about their health; different ways participants would use or not use the QPLs offered; and, varied levels of interest or endorsement of the use of QPLs. As each participant was interviewed on average three times a relationship was developed to a varying extent between participant and interviewer. This relationship was utilized to explore values and perceptions of the tools and interactions participants had with the tools and their HCPs. Notes from previous interviews were reviewed before the next interview with that participant. At all stages before, during and after data collection, the implications of the interviewer's role were discussed among the research team.

### 2.7 Role of the funding sources

Our funders played no role in the design of the study.

### 2.8 Reference group

The reference group associated with this study had representatives from the Australian Commission on Safety and Quality in Health Care, Healthdirect Australia Ltd., consumer representation and study team members (M. T., L. T. and H. L.). The group met quarterly and was consulted on the draft protocol and briefed on the findings of the study. The final design and dissemination of findings were the sole responsibility of the research team.

## 3 RESULTS

The recruitment strategy resulted in 300 people accessing the advertisement links and 124 completed eligibility surveys (Table 2). From these, 32 people were purposively chosen and invited to participate in an interview (one declined) (Appendix SB). Participants who were not invited were sent an email thanking them for their interest and participation in the study. After 31 first interviews, the authors considered that the sample had adequate diversity and that there was sufficient breadth and depth of data to answer the research questions (Table 2). The study interviews included a mixture of participants who had planned medical appointments, unplanned visits and those who had no appointments during the study. The average interview length was 23 min.

**Table 2:** Summary of interview and completed survey participant demographics

Demographic	Interview participants (*n* = 31 [%])	Completed survey (*n* = 124 [%])
Age range	20–77 years (mean: 46.3)	19–91 (mean: 46.7)
Gender identification	15 male, 16 female	34 male, 90 female
Identified as Aboriginal Australian^a^	2 [6]	5 [4]
Highest education level attained		
University degree	19 [62]	81 [65]
Diploma/certificate	6 [19]	28 [23]
Trade apprenticeship	0 [0]	1 [1]
Higher school certificate or leaving certificate	5 [16]	11 [9]
School certificate or intermediate certificate	1 [3]	3 [2]
Basic health literacy scores (/15)^b^	10–15 (mean: 13.4)	9–15 (mean: 14)
Level of patient activation (CHAI score)^c^		
Low (<80)	21 [68]	73 [59]
Moderate (80–94)	10 [32]	43 [35]
High (>95)	0 [0]	8 [6]
Speaks a language other than English at home	8 [26]	17 [14]
Chronic health condition^d^	22 [71]	79 [64]
Carer^e^	5 [16]	19 [15]
*Place of residence*		
State or Territory (% of the Australian population)^f^		
Australian Capital Territory (2)	1 [3]	5 [4]
New South Wales (32)	9 [29]	57 [46]
Northern Territory (1)	1 [3]	1 [1]
Queensland (20)	2 [6]	17 [14]
South Australia (7)	2 [6]	8 [6]
Tasmania (2)	1 [3]	3 [2]
Victoria (26)	9 [29]	24 [19]
Western Australia (10)	1 [3]	8 [6]
Remoteness		
Major city	21 [68]	90 [72]
Inner regional	4 [13]	23 [18]
Outer regional	6 [19]	10 [8]
Remote	0 [0]	1 [0]
Recruitment source		
Question Builder	0	0
Symptom Checker	26 [84]	111 [90]
HealthDirect Facebook	5 [16]	13 [10]
Number of interviews conducted with each participant		
1	2^g^	n/a
2	0	n/a
3	26	n/a
4	3^h^	n/a

Abbreviation: n/a, not available.

^a^ Aboriginal and Torres Strait Islander identifying people are approximately 3.3% of the total Australian population.^35^

^b^ Chew et al.,^30^ Basic Health Literacy Score (BHLS) screening tool, low health literacy defined as score ≤9.

^c^ Consumer Health Activation Index (CHAI) score.^31^

^d^ Participant self‐identified as having a condition lasting more than 3 months in the participant survey (‘Do you have a health condition which has lasted more than 3 months?’).

^e^ Participant self‐identified as being a carer in the participant survey (‘Do you have someone who is dependent on you for their daily care needs?’).

^f^ Australian Bureau of Statistics Population data at 31 December 2020.^36^

^g^ One participant lost to follow‐up, and one participant opted out of the study.

^h^ Three participants were interviewed a fourth time as they had an imminent medical appointment booked within a short time of their third interview.

### 3.1 Themes

Participant quotes are identified by: (participant number_interview number), for example (4_2) is a quote from participant four in their second interview (Table 3 shows individual participant data).

**Table 3:** Individual participant demographic descriptions

Participant number	Age range (years)	Identified gender	Level of patient activation (CHAI score)	Health literacy score (/15)	Number of interviews
1	31–60	F	83	15	3
2	18–30	M	50	13	3
3	31–60	M	90	12	3
4	18–30	F	58	15	3
5	31–60	M	63	10	1 (Opted out)
6	31–60	F	92	15	3
7	18–30	F	88	13	3
8	18–30	F	80	11	3
9	>60	M	72	15	4
10	>60	F	48	10	3
11	18–30	F	72	15	3
12	18–30	M	92	13	3
13	18–30	F	93	10	3
14	>60	F	92	15	3
15	31–60	F	57	14	3
16	31–60	M	60	15	3
17	31–60	M	57	12	3
18	31–60	F	48	15	1 (Lost to follow‐up)
19	31–60	M	57	14	3
20	31–60	M	65	11	3
21	>60	F	80	15	4
22	31–60	F	57	15	3
23	31–60	M	57	15	3
24	31–60	M	75	15	4
25	31–60	M	63	13	3
26	18–30	M	50	13	3
27	18–30	F	67	13	3
28	31–60	F	78	15	3
29	31–60	M	62	11	3
30	31–60	F	93	15	3
31	>60	F	70	12	3

Abbreviations: F, female; M, male.

Interview participants responded to the semi‐structured interview questions, mostly seeking health information by not only sharing their experiences but also frequently revealing further contextual and personal information. Some participants with chronic health conditions had extensive interactions with the health system over many years, others described life‐changing interactions in acute health crises experienced by themselves or a family member, several revealed they were trained health professionals (nurses, paramedics) and some participants had very little experience with the Australian health system.

Three themes were developed: First describes the difficulties many participants face asking questions and accessing health information; second focuses on the responses of participants to the QPLs and using them in practice, while the third theme describes suggestions for improving the QPLs.

#### 3.1.1 Theme 1: There are many factors influencing patients' access to health information

Barriers and facilitators were described by participants in their journeys to access health information.

**1.1: ‘It was a system that I was not confident navigating and had no experience with’ (4_1)**

Participants revealed varying levels of experience with the health system. Many described the complexity of the system in which they were trying to seek information about their health.

*People sometimes avoid going to get medical advice because it's just too difficult*. (23_1)

Time and financial constraints further compound the issues of navigating the health system to get the information participants were seeking. Sourcing information for participants could be time‐consuming and frustrating.

*So, when I have questions, I probably ‐ I'm not going to go back to the GP, because I have to go and sit there for half an hour and they're always running late, and I can't be bothered…*. (30_2)

**1.2: Asking, and knowing to ask questions is hard**

For some participants, there was uncertainty about whether to ask a question, what to ask and how to ask:

*Yeah, that's my other problem. It's trying to figure out what the questions are or how to ask them*. (24_3)

*“I don't know how to ask things, so I don't*. (10_1)

Many factors impacted participants' comfort with asking questions: their medical issue; perceived doctor engagement ‘I didn't feel that they were that interested’ (19_2); whether it ‘was a bit scary’ (13_1); or ‘It feels rushed’ (27_1). Conversely, question‐asking was more comfortable when doctors ‘don't make you feel rushed’ (4_1).

Some perceived a power imbalance and did not want to waste the doctor's time. This also influenced participants' decisions about asking questions.

*I don't want to have to push in and ask questions…*. (18_1)

In contrast, other participants knew what information they required and would ‘keep on asking questions until I feel satisfied’. (16_2).

**1.3: ‘I think someone that can talk to a patient is the best’ (21_1)**

This subtheme explores the various characteristics of health professionals, particularly doctors, which participants valued highly and helped them get the information they wanted or needed. Participants wanted a doctor who was ‘happy to sit there and talk to me’ (22_1) for ‘more of a two‐way discussion’ (23_1) and a ‘human exchange’ (23_1), but also described the importance of how a doctor should ‘never (make you) feel as though you are asking a stupid question’ (6_1).

Participants provided the following descriptions of doctors' behaviours and attitudes that assisted them to ask questions and access health information: ‘Someone that listens to you’ (15_1); a doctor who makes them ‘feel confident in asking questions’ (23_3); ‘Always willing to answer any questions’ (4_2); ‘Should have quite ample communication skills’ (1_1); ‘Sit(s) back and absorb(s) everything you've asked’ (18_1); Explains ‘it pretty thoroughly’ (4_1); ‘Provide(s) very clear answers’ (11_1), ‘in very simple terms'’ (3_1) and avoids ‘clinical language’ (24_4); Gives ‘a lot of options to decide what the next steps should look like’ (3_1) and should not be ‘reluctant to admit’ (19_1) if they don't know what is going on.

#### 3.1.2 Theme 2: Generic QPLs have many benefits—Changing attitudes and behaviours (or not)

This theme focuses on participants' thoughts and feelings when using one or both generic QPL tools—QB and ASK.

**2.1: Perceived role of question‐asking in consultations**

Participants described how exposure to the QPL tools normalized question‐asking, ‘I don't have the worry of… am I wasting this doctor's time’ (3_3). Others described how the tools were useful ‘as well as giving (me) permission’ (10_2) to ask questions to access information. For some, the QPLs increased confidence in expressing the questions they wanted to ask and gave them what they felt were the right words:

*…maybe the way it was written (ASK), or ‐ it just made me put the questions in my head a little bit better*. (10_2)

A few participants expressed uncertainty about using the tools and were *‘not sure if I ever understood the idea of the three questions’*. (4_3)

For others, it was familiarization, engagement and putting the tool into practice in consultations that opened new experiences of receiving information and discovering they had options. This increased awareness of wanting and needing more information:

*They were things that came up as a result of me asking a question. She would explain something, and I'd ask another question about it, like it would bring up a whole different set of things, set of options that might be available. So, I didn't anticipate those options being available and then I had a couple of questions about that which was why I'm seeing her again*. (4_2) 

Having their doctor mention options to them in response to asking questions meant some participants then realized that there was a choice involved and that the choice could be made by them:

*So, I never really had that feeling of yes, a choice of a treatment is mine. So, this time it felt good to have that*. (11_2)

Participants more experienced at asking questions did not explicitly discuss using the QPLs in consultations because they were, ‘already doing that (asking questions about options)’ (15_3).

**2.2: Power and empowerment**

Highly engaged and self‐reported experienced question‐askers expressed some vindication of their current approach, although they thought the tools were good for others with less experience:

*I know that you have options. I still found it useful to have it laid out like that, because yes, that's something I can tell other people. You've got options. It's your decision with some guidance*. (21_2)

In contrast, those with less experience ‘felt more… empowered in a way, that I could be more involved in the health decisions that I'm making… and be more informed’ (7_2). While there were other participants who did not think SDM and question‐asking were necessary for their health journey:

*…the reality is, I'd still be looking for the doctor to go, this is my recommendation, and I'd probably say – nine times out of ten I'd probably be just saying, doctor knows best*. (29_3)

**2.3: Preparation, prioritization and agenda‐setting**

This subtheme explores participants' behaviours and attitudes to prepare for interactions with health professionals after exposure to QPLs. Again, participants' previous experience influenced the impact of QPLs on attitudes and behaviours. Participants with higher health literacy and more experience with the health system felt there was a less notable use for the tools; however, they found them easy to use:

*I probably wouldn't use it because I feel competent enough to ask the right questions*. (13_3)

*Yeah, it was easy to follow for me, because I've had a lot of experience with specialists*. (1_2)

The QPLs reinforced their usual practices with a little more structure:

*Having that sort of approach… makes a massive difference*. (15_3)

However, some participants who had ‘never really even thought about the idea of having really structured questions’, (4_3) saw the possibilities in QPLs to ‘prepare you, I guess’ (26_4) for a consultation.

Some participants perceived that prioritizing questions helped clarify what was important to them:

*I think the three (ASK) questions would really help laying out like a framework of potential things I could ask or get responses to*. (28_2)

Identifying which questions to prioritize was challenging for some:

*I picked most of the questions, I thought, they're all relevant. … But then when you're asked to prioritise those there's no criteria to base your decision on*. (16_3)

Participants recognized that using QPLs to prepare for consultations facilitated getting the most out of consultations, including information that was most useful to them for SDM:

*It definitely helped guide me, get more information and make more informed decisions*. (7_2)

#### 3.1.3 Theme 3: It would be good if…

With so many combined experiences of using QPLs, participants had lots of thoughts about how QPLs could be improved.

**3.1: QPLs could be easier to use**

There were many QPL features that worked well for participants:

*…your tool's plain English*. (3_3)

*…I just went straight in and started using it*. (21_3)

However, some found instructions for ‘both of them were a little bit confusing but (I) then got around it’ (8_3) and that, ‘There was a lot of words, and I think I get the gist of it’ (2_2).

Several participants commented on the number of questions (191) in QB or that taking a physically large list of questions was not very feasible:

*The only thing I do remember is thinking that there's too many questions…There's too many to look at. It's overwhelming*. (25_3)

*Question Builder…there's so many…questions. …I had four sheets of paper which I wasn't happy with*. (9_4)

**3.2: QPLs should be where question‐asking happens**

To be most useful, participants described how QPLs should be easy to find when they needed them. Several participants brought up the idea of the tools being available as an application for their phones.

*I think an app would actually be much more useful and accessible as well… I would want it to have features to be able to take notes in response to the questions*. (7_2)

Another popular idea was for QPLs to be linked to appointment software systems to suggest you prepare for the consultation, or a link sent via SMS if booking directly through the practice. Many noted that leaflets or posters in the waiting room would be good prompts to use the tools.

*So, it has to be easily accessible at the time you need it. I think that's probably my key thing, so I don't know whether it's just something in the waiting room, or when you make a booking online*. (30_3)

**3.3: QPLs and QA need to be normalized for doctors, patients and the health system**

A recurring sentiment throughout the interviews was that question‐asking and using a prompt list if required should be standard practice; ‘It can't just be up to the individual to ask these questions’ (23_3).

*If that was the sort of behavioural norm, I guess, as in go to your GP, have these three things… If it became just part of,…the common practice…*. (29_2)

During the study, no participants reported negative responses from their HCP to them asking questions. Even so, participants wanted their doctor's endorsement of question‐asking and the QPL tools to make them feel more comfortable using them.

*…maybe I'd have a look at it because I'd be like oh well the doctor recommended it*. (13_3)

And finally, there were participants who thought education was an important factor in normalizing question‐asking and QPLs.

*AskShareKnow now looking at it would be something that would be fantastic if they actually put this in schools*. (23_3)

## 4 DISCUSSION

Our results support data that there are significant challenges navigating a complex health system for people seeking health information and preparing for consultations.^23^ To our knowledge these are the first published data evaluating the use of currently available generic QPLs in real‐world settings. Participants had different past experiences and knowledge of the health system. There were several participants who described being hesitant to ask questions unless the doctor or other HCP provided a comfortable and encouraging environment to do so. Generic QPLs normalized the concept of question‐asking and helped those with less experience to overcome their uncertainties. Participants reported that using QPLs ‘empowered’ them and helped them better prepare for appointments with their doctor. Endorsement and the normalization of question‐asking plus having easy‐to‐use QPLs at first contact with clinical settings were recommended by participants.

Frosch et al.^37^ described strikingly similar patient attitudes to asking questions in consultations; patients didn't want to appear ‘difficult’, they avoided asking questions and described additional work outside consultations to meet their information needs. This study adds that QPLs can positively influence behaviours and perceptions towards asking questions and SDM for some people.

Australian Government health literacy data from a 2018 survey, which used a long health literacy questionnaire, found 8% of people had difficulty ‘understanding health information well enough to know what to do’.^23^ While no direct correlation can be made between the BHLS screening tool^28^ scores and Australian health literacy data we had 2 of the 31 (6%) participants who scored themselves on the lowest or second‐lowest rating on at least one question compared with 8% in the Australian data on similar domain questions.^23^, ^38^, ^39^ This study provides data to assist in understanding the experiences of using QPLs from participants who reported a range of levels of health literacy and activation for future design and implementation of QPLs.

There were marked differences in the impact of QPLs on participants' attitudes and behaviours towards question‐asking depending on their self‐described past experiences as a patient and level of health knowledge. Participants' experience ranged from those who had significant health care knowledge through experiences or their occupation (e.g., nurse, paramedic) and those with almost no independent use of the health system. In addition, as found in previous research, participants with lower health literacy levels generally experienced the greatest benefits in terms of empowerment and understanding of the role of question‐asking in consultations.^40^ Our results support previous findings that QPLs may facilitate SDM through improving health literacy and person‐centred communication.^41^ Further, this improvement in health literacy may also assist people to access health services and address some of the inequities in health care provision and outcomes.^42^

### 4.1 Strengths and weaknesses

Our study sample was very diverse but solely sourced online due to COVID‐19 restrictions. Online QPLs can be introduced to patients by other means, such as waiting room posters, however, due to COVID‐19 we were unable to assess the attitudes of any people who may not be as experienced with online environments. A strength of our sample was that it included those with low‐moderate patient activation, a variety of levels of education, a broad range of ages and locations by rurality and jurisdiction in Australia. Further, while health literacy screeners indicate those with very low health literacy levels, our sample included participants who found challenges in understanding health information, even if the health literacy screener was not sensitive enough to pick this up.^43^

The study type used was a longitudinal qualitative study. Patient journey methodologies can be used to provide ‘symbolic support for the principles of PCC’ even though data as a tool for health service accreditation of person‐centred care are still lacking.^44^ For this study we followed patients in their usual health care with the addition of QPLs and recorded their experiences. While this is an uncommon study type it allowed participants to have time to consider and review each tool and increase the chance that they would be able to use them in consultation and provide details of their experiences.

All interviews were conducted by a single researcher (M. T.). This allowed continuity across interviews and for participants to pick up where the last interview had finished. Participants were aware that neither tool had been developed by M. T. and participants were given access to the QPL tools but no further instructions about whether or how to use them. We acknowledge that demand characteristics are a consideration in the interpretation of the results of the study and that participants may have been more positive about the tools and experiences than they genuinely felt.

It is possible that even though the intervention was exposure to the tool and not an instruction to use the tool, some participants may have felt uneasy using the tools or had other unintended consequences. No participants reported to the study investigator any adverse experiences as a direct result of the study. A few participants mentioned significant previous health events while describing past instances of accessing health information. All participants were reminded that detail of their medical history was not required, nor would it be reported. Assessing the clinical relevance of the questions asked, the detail of any information received, or health outcomes related to QPLs was outside the scope of this paper but warrants future research.

## 5 CONCLUSION

This study shows that the introduction of QPLs can improve knowledge of how, when and why question‐asking is a helpful part of medical consultations and that they can encourage patient engagement. To maximise benefits and normalise use of these tools, supporting patient question‐asking needs to be part of education for healthcare providers, best‐practice healthcare standards and guidelines. QPLs need to have clear instructions, be endorsed by HCPs and be readily available in online and physical locations where people seek answers to healthcare questions. Future research should evaluate the implementation of point of care delivery of generic QPLs, noting that AHRQ has created an app version of QB.^45^ Most importantly, endorsement of question‐asking and QPLs by medical practitioners, and in all parts of health systems is essential to their effectiveness.

## AUTHOR CONTRIBUTIONS

Marguerite Tracy, Heather Shepherd, Emerita Lyndal Trevena conceived the study, its design and methods. Olivia Mac assisted in organizing participant data, REDCap survey data and transcription files. Marguerite Tracy conducted all participant interviews. Initial coding was performed by Marguerite Tracy and Julie Ayre to establish codes. Marguerite Tracy, Olivia Mac, Julie Ayre, Tessa Copp, Heather Shepherd and Emerita L. Trevena coded transcripts, as well as discussed and agreed on codes and themes. Marguerite Tracy drafted the manuscript, Olivia Mac, Julie Ayre, Tessa Copp, Heather Shepherd and Emerita L. Trevena reviewed drafts and agreed on the final manuscript.

## CONFLICTS OF INTEREST

The authors declare no conflicts of interest.

## Supporting information

Supplementary information.

Supplementary information.

## Data Availability

The data that support the findings of this study are available on request from the corresponding author. The data are not publicly available due to privacy or ethical restrictions.
