# Supplementary material for: Question prompt lists and endorsement of question‐asking support patients to get the information they seek—A longitudinal qualitative study
Source: Health Expect. 2022 Apr 26;25(4):1652–63. doi: 10.1111/hex.13509 (PMC9327807; doi:10.1111/hex.13509)
Supplement: Supplementary file 1 — Supplementary information. [file HEX-25--s002.docx]

Appendix 1: Interview guides

There are three guides below for each of the three interviews conducted with participants of the study.

_______________________________________________________

**Patient Journeys to Health Information**

Semi-structured interview guide

Initial interview

“We are interested in talking to people about how they find answers to questions about their health. Do you find that you often have questions about your health, or your family’s health?”

**“Can you tell me about a time when you** looked **for information about your health, or had a question about your health (or the person you care for/or your family’s health)?”**

Possible prompts:

Can you tell me a bit more about how you found your answer?

Can you explain the steps you might take to find that information?

What do you think about the different sources of information you use/ have used in the past?

How would you like to be able to get answers about your health?

How do you feel about how likely you are to get information about your health?

How does it make you feel when you are finding it difficult to get information?

What sources do you use to get information about your health?

What tools have you used, or do you use to help you get health information?

Can you describe the sort of information you have found/received?

**“Can you tell me about a time when you asked a health professional questions about your health/your child’s health/ the person you care for’s health?”**

Possible prompts:

Can you explain how you ask questions when you see a health professional?

What have the experiences of asking them questions been like for you?

What do you think about the information you have received from your health professional?

How do think health professionals might be able to help you get the health information you need?

How do you feel about asking your health professional questions?

How does it make you feel when you are finding it difficult to get health information?

How would you describe health professionals’ willingness to be asked questions?

What tools have you used/ are aware of/ do you use to help you get health information from your health professional?

In general, how do you feel about asking your health professional questions?

Can you describe how it actually feels to ask your health professional questions?

Which health professionals do you ask for information about your health?

How do you see your role in the consultation?

Do you think your doctor expects you to ask questions?

For the first call:

Participants will then be offered access to ASK, Question Builder, both or neither and told how to find the resources while on the call.

Participants will then be asked if they have a medical appointment booked/planned in the next month. If so, then the follow up phone call will be organised as soon as possible after the appointment. Participants will be asked to notify the researcher if they attend an unplanned appointment. If neither occurs a future appointment will be made for one month’s time.

Give link to QB/ASK

Organise next meeting – if doctor’s appointment – asap following appointment

_______________________________________________________

**Patient Journeys to Health Information**

Semi-structured interview guide

Follow up interview 1

Introduction

- Introduce self, ensure comfort of interviewee and that they have time to talk.
- Reiterate consent (over 18) and re-explain the nature of the discussion, timeframe and duration of interviews and again confirm they still wish to participate.
- Remind again that the interviews are being recorded and that they can withdraw from the study at any time.
- Remind, as required, that any medical conditions, medical information about themselves, or anyone they care for, or the actual information they are seeking, does not need to be disclosed at any time during the study. It is preferable for them to be non-specific about the health information they seek.

Confirm whether they have had a recent consultation with a health professional.

Yes:

- **Can you tell me about whether you had any questions you wanted to ask to ask your doctor at your recent appointment?**
- (If they did) **Can you tell me about how you did, (or didn’t), get the information you wanted?**
- During the consultation how did the amount of information you wanted or the number of questions you wanted to ask change?

Sample prompts:

- How did you feel about asking questions in that consultation?
- Ask if they accessed the question prompt list tool/s.
- Any issues accessing the tool?
- What were your initial thoughts about the tool?/ What did you hope that the tool/s would be able to do for you?
- Can you tell me about how many questions you asked/ wanted to ask?
- How did the (ASK/QB) questions influence how you asked/didn’t ask questions in the consultation?
- Paper or phone list?
- How did the doctor respond to your questions/ the QPL?
- How has QB/ ASK altered how you feel about asking questions when you go to the doctor?
- How was the information different to information you have had in the past?
- How did you feel about the consultation when you left?
- What would you do differently before or during your next appointment with that doctor or a different doctor?
- Who do you think would find this QPL useful?

No: If no recent consultation:

- Since we last spoke have you had new questions about your health or healthcare?
  - Yes – can you tell me about how you tried to answer your question/s?
  - No- go to questions about tool:
- Ask if they accessed the question prompt list tool/s.
- Any issues accessing the tool?
- What were your initial thoughts about the tool?/ What did you hope that the tool/s would be able to do for you?
- How might this tool be useful to you?
- **In what ways were you able to use the tool/s?**
- Would it make a difference if the doctor had said to use the tool?

Participants will then be offered access to ASK, Question Builder, or neither depending on what they have already been exposed to and told how to find the resources while on the call and sent link following the call.

The final follow up call will be booked with the participant for approximately two month’s time, or immediately after medical appointment, whichever is earlier .

_______________________________________________________

**Patient Journeys to Health Information**

Semi-structured interview guide

Follow up interview 2

Introduction

- Introduce self, ensure comfort of interviewee and that they have time to talk.
- Reiterate consent (over 18) and re-explain the nature of the discussion, timeframe and duration of interviews and again confirm they still wish to participate.
- Remind again that the interviews are being recorded and that they can withdraw from the study at any time.
- Remind, as required, that any medical conditions, medical information about themselves, or anyone they care for, or the actual information they are seeking, does not need to be disclosed at any time during the study. It is preferable for them to be non-specific about the health information they seek.

**Confirm whether they have had a recent consultation with a health professional.**

Yes:

- **Can you tell me about whether you had any questions you wanted to ask to ask your doctor at your recent appointment?**
- (If they did) **Can you tell me about how you did, (or didn’t), get the information you wanted?**
- During the consultation how did the amount of information you wanted or the number of questions you wanted to ask change?

(If no to these questions then explore thoughts about the tools, other sources of information, potential barriers to question asking, ; If tools not accessed ask about potential the barriers to access...

Sample prompts:

Before your appointment:

- Did you manage to access QB / ASK?
- Any issues accessing the tool?
- What were your initial thoughts about QB / ASK?/ What did you hope that QB / ASK would be able to do for you?
- (If applicable) Which of the two tools/ question lists did you prefer and why?
- How did you decide what you wanted to ask? QB - Did you prioritise any questions?
- Could you share what types of questions you chose/wanted to ask?
- Did you do anything else before the appointment to find an answer to your question? - Did you seek information elsewhere? Did you create your own list of problems/questions for the appointment?

During the appointment:

- Can you tell me about how many questions you asked/ wanted to ask?
- **How did you feel about asking questions in that consultation?**
- Was there a part of the appointment where it felt like the time to ask questions? - explore this
- How did the (ASK/QB) questions influence how you asked/didn’t ask questions in the consultation?
- Paper or phone list?
- How did the doctor respond to your questions/ the QPL?
- How has QB/ ASK altered how you feel about asking questions when you go to the doctor?
- How was the information different to information you have had in the past?
- If you didn’t use QB/ASK – how did you get information from the doctor you needed?

After the appointment:

- How did you feel about the consultation when you left?
- What would you do differently before or during your next appointment with that doctor or a different doctor?
- Who do you think would find this QPL useful?
- Which one would you be more likely to use in future?
- How would you feel if the doctor told you about QB / ASK?
- How would you feel if there was not enough time for your questions?
- What would you do if there was not enough time for your questions?

No: If no recent consultation:

- **Since we last spoke have you had new questions about your health or healthcare**?
  - Yes – can you tell me about how you tried to answer your question/s?
    - what types of questions?
  - No- go to questions about tool:
- Did you manage to access QB / ASK?
- Any issues accessing the tool?
- What were your initial thoughts about QB / ASK?/ What did you hope that QB / ASK would be able to do for you?
- (If applicable) Which of the two tools/ question lists did you prefer and why?
- How might this tool be useful to you?
- Who do you think would find this QPL useful?
- Which one would you be more likely to use in future?
- **In what ways were you able to use the tool/s?**
- How would you feel if your doctor told you about QB / ASK?
- How would you feel if you did not have an opportunity to ask your question/s?
- Where would you expect to find them so they could be useful to you?

Do you have a consultation/appointment planned with a doctor soon?

Yes:

- Do you have any questions that you are planning to ask your doctor?
- Can you tell me about how you would get ready to ask questions?
- How might you use QB / ASK before your next appointment?
- What benefits might there be?
- What issues might you face using QB / ASK?
- How would your doctor’s response to your questions affect you?

How do you see your role in the consultation?

Do you think your doctor expects you to ask questions?

No: End of interview.

- Thank participant for their time and their participation in our research
- Remind them to look out for an email about the final voucher
- Let them know they can contact us if they have any concerns or questions and that they will receive a copy of the summary of our findings at the end of the study.

_______________________________________________________
