# Supplementary material for: Question prompt lists and endorsement of question‐asking support patients to get the information they seek—A longitudinal qualitative study
Source: Health Expect. 2022 Apr 26;25(4):1652–63. doi: 10.1111/hex.13509 (PMC9327807; doi:10.1111/hex.13509)
Supplement: Supplementary file 2 — Supplementary information. [file HEX-25--s001.pdf]

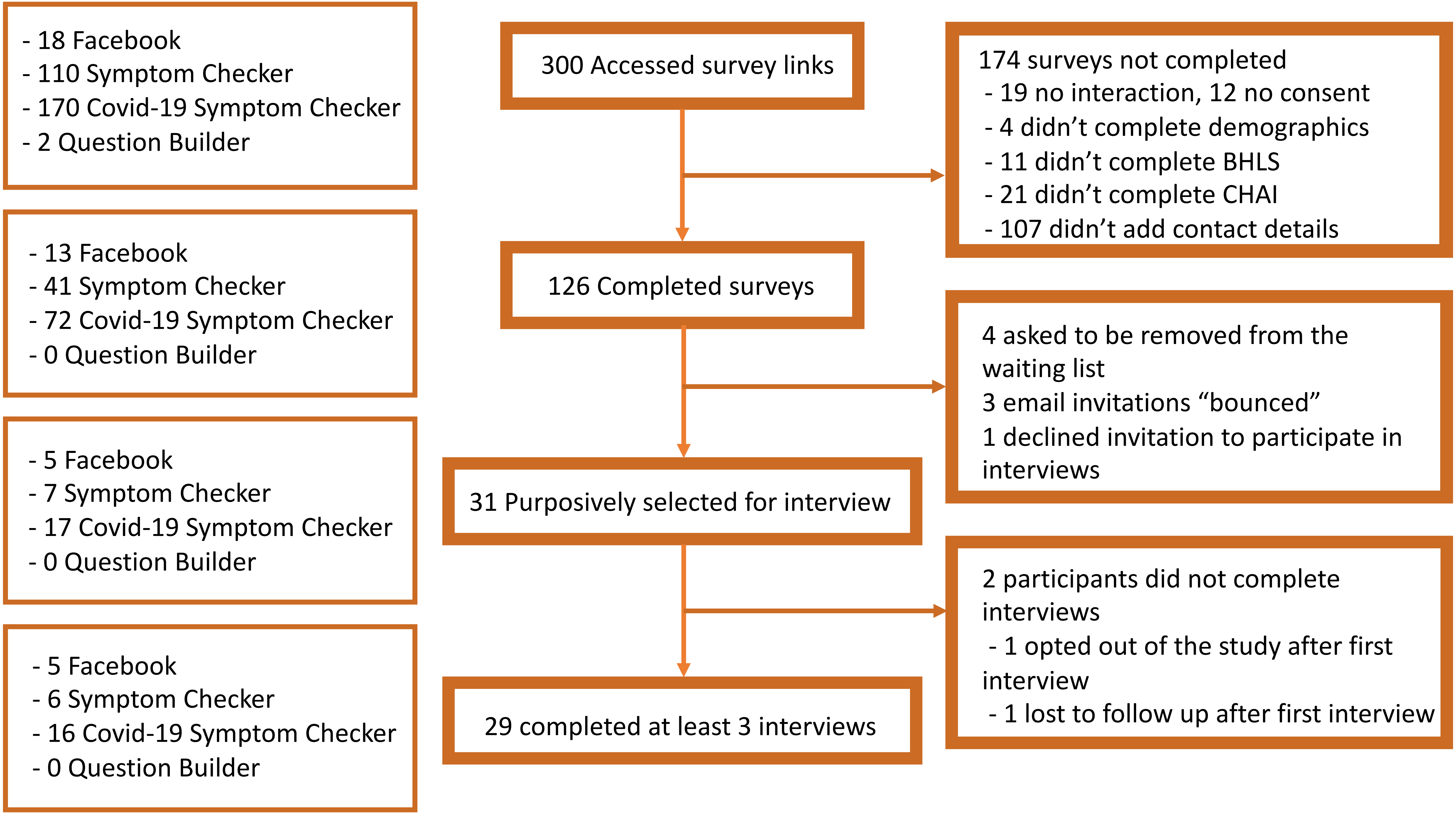
- 18 Facebook
- 110 Symptom Checker
- 170 Covid-19 Symptom Checker
- 2 Question Builder
- 13 Facebook
- 41 Symptom Checker
- 72 Covid-19 Symptom Checker
- 0 Question Builder
- 5 Facebook
- 7 Symptom Checker
- 17 Covid-19 Symptom Checker
- 0 Question Builder
- 5 Facebook
- 6 Symptom Checker
- 16 Covid-19 Symptom Checker
- 0 Question Builder
300 Accessed survey links
126 Completed surveys
31 Purposively selected for interview
29 completed at least 3 interviews
174 surveys not completed
- 19 no interaction, 12 no consent
- 4 didn't complete demographics
- 11 didn't complete BHLS
- 21 didn't complete CHAI
- 107 didn't add contact details
4 asked to be removed from the waiting list
3 email invitations "bounced"
1 declined invitation to participate in interviews
2 participants did not complete interviews
- 1 opted out of the study after first interview
- 1 lost to follow up after first interview
